# Recent Advances on Stimuli-Responsive Hydrogels Based on Tissue-Derived ECMs and Their Components: Towards Improving Functionality for Tissue Engineering and Controlled Drug Delivery

**DOI:** 10.3390/polym13193263

**Published:** 2021-09-25

**Authors:** Julian A. Serna, Laura Rueda-Gensini, Daniela N. Céspedes-Valenzuela, Javier Cifuentes, Juan C. Cruz, Carolina Muñoz-Camargo

**Affiliations:** Department of Biomedical Engineering, Universidad de los Andes, Bogotá 111711, Colombia; ja.serna10@uniandes.edu.co (J.A.S.); l.ruedag@uniandes.edu.co (L.R.-G.); dn.cespedes@uniandes.edu.co (D.N.C.-V.); jf.cifuentes10@uniandes.edu.co (J.C.)

**Keywords:** extracellular matrix, hydrogels, external stimuli, tissue maturation, drug delivery

## Abstract

Due to their highly hydrophilic nature and compositional versatility, hydrogels have assumed a protagonic role in the development of physiologically relevant tissues for several biomedical applications, such as in vivo tissue replacement or regeneration and in vitro disease modeling. By forming interconnected polymeric networks, hydrogels can be loaded with therapeutic agents, small molecules, or cells to deliver them locally to specific tissues or act as scaffolds for hosting cellular development. Hydrogels derived from decellularized extracellular matrices (dECMs), in particular, have gained significant attention in the fields of tissue engineering and regenerative medicine due to their inherently high biomimetic capabilities and endowment of a wide variety of bioactive cues capable of directing cellular behavior. However, these hydrogels often exhibit poor mechanical stability, and their biological properties alone are not enough to direct the development of tissue constructs with functional phenotypes. This review highlights the different ways in which external stimuli (e.g., light, thermal, mechanical, electric, magnetic, and acoustic) have been employed to improve the performance of dECM-based hydrogels for tissue engineering and regenerative medicine applications. Specifically, we outline how these stimuli have been implemented to improve their mechanical stability, tune their microarchitectural characteristics, facilitate tissue morphogenesis and enable precise control of drug release profiles. The strategic coupling of the bioactive features of dECM-based hydrogels with these stimulation schemes grants considerable advances in the development of functional hydrogels for a wide variety of applications within these fields.

## 1. Introduction

Tissue engineering (TE) and regenerative medicine (RM) are closely related research fields where biology, medicine, and engineering converge towards the development of solutions for in vitro or clinical applications. The most recurrent include repairing or replacing tissues whose function is impaired, and developing relevant tissue models for testing drugs or studying pertinent mechanisms of prevalent and rare diseases [1]. Over the past 20 years, hydrogels have been key to the great advances made in these fields to manufacture functional tissue-like structures and the implementation of minimally invasive therapeutics for regenerative and drug delivery purposes. In particular, their highly hydrophilic nature and compositional versatility makes them ideal building blocks for designing cell-friendly and multifunctional microenvironments suitable for engineering tissues [2].

Recent efforts have been focused on designing hydrogels whose physicochemical and mechanical characteristics are ideal for their use as enablers of currently emerging biofabrication technologies [3]. This field focuses on the automated generation of structurally organized and biologically functional products for TE and RM applications. The main goal of this approach is to develop in vitro tissue models or transplantable constructs for in vivo repairing or replacement of tissues [4]. Among the ample variety of 3D bioprinting techniques, the extrusion-based ones have proven the most popular and with the highest potential to fabricate anatomically relevant, multi-material, tissue-like constructs [5]. Hydrogels suitable for extrusion-based 3D bioprinting should exhibit physical properties such that they can be extruded through a nozzle or needle in a controlled manner without losing their integrity (i.e., printability) [6,7,8]. This can be accomplished by properly tuning their mechanical properties, specifically their rheological behavior and viscosity profiles. In this regard, mechanically robust hydrogels must be viscous enough to be able to form a filament as they are extruded and deposited on a surface, and chemically suited to build structurally sTable 3D constructs upon crosslinking [9,10,11]. Another relevant application of hydrogels is in drug delivery, where they have been widely implemented as depots for the localized and controlled release of therapeutic molecules, growth factors, and cells [12,13,14]. In particular, the encapsulation of these agents within hydrogels provides improved bioavailability, high and controlled release rates, and protection from early enzymatic degradation. This makes hydrogels powerful carriers for developing highly biocompatible and tunable delivery systems for the treatment of various diseases [15,16].

Depending on their origin, the materials commonly employed in the formulation of hydrogels can be classified into either synthetic or natural. Synthetic materials are known to have exceptional mechanical and physicochemical properties, but lack the bioactivity exhibited by natural materials [2]. Recently, hydrogels derived from decellularized extracellular matrices (dECMs) have been explored as the most compelling candidates for mimicking the microenvironments of native tissues [17,18,19]. This has been attributed to the fact that, unlike other natural or synthetic materials, extracellular matrices (ECMs) exhibit a cocktail of bioactive molecules that interact synergistically to create the adequate environment for cellular development. ECMs hold a complex of hierarchically assembled fibrous proteins that comprise the structural backbone of tissues and grant adequate mechanical stability for supporting cellular adhesion, chemotaxis, and migration in vivo [20]. The most abundant of these fibrous proteins are various types of collagen, which are the main structural components of ECMs due to their ability to self-assemble into hierarchically organized fibers or networks [21]. These collagen structures are responsible for providing stiffness and tensile strength, as well as for most of the mechanotransduction cues produced during tissue development [22]. Moreover, different types of collagen can also interact with cells through membrane receptors, thus actively participating in cell growth, differentiation, and migration [23]. Other fibrous proteins, such as elastin and fibronectin, associate with collagen structures to counter their characteristic stiffness and are responsible for tissue elasticity and mechano-regulation. In turn, the relative ratio of these proteins within ECMs yields different mechanical characteristics, which are closely related to native tissue functionality [20]. Another large family of ECM molecules are proteoglycans, which comprise glycosaminoglycan (GAG) chains covalently linked to a protein core. An exception to this is hyaluronic acid (HA), a high-molecular weight GAG that, instead, interacts noncovalently with ECM proteins [24]. GAGs are extremely hydrophilic and adopt extended conformations essential for hydrogel formation and for withstanding high compressive forces [25]. Accordingly, this hydrated network of GAGs and proteoglycans comprises the majority of the extracellular space and closely interacts with fibrous proteins to create a versatile network for cellular scaffolding. As several of the growth factors secreted by cells can bind to their surrounding matrix, an easily accessible repository for such factors is enabled by this hydrated network. This ultimately helps direct essential morphological organization and physiological function [20]. Accordingly, the heterogeneous composition of ECMs, rich in essential molecules implicated in native tissue development, makes them ideal materials for the development of hydrogels intended for TE and RM applications. dECM-based hydrogel formulations are, therefore, not only biocompatible, but also provide superior bioactive cues for guiding cellular behavior when compared to other natural materials [26].

To isolate ECMs and formulate hydrogels with them, tissue samples are exposed to decellularization procedures and subsequently solubilized to form pre-gels, which conserve the majority of the original ECM components and can later be patterned into constructs with desired geometries [27]. However, decellularization and solubilization procedures involve mechanical and chemical processing, as well as the use of proteolytic enzymes, which largely disrupt the hierarchical organization of ECM components and alter their native architecture [27]. Although the components of dECM hydrogels have been shown to partially re-assemble under physiological conditions (i.e., 37 °C, pH 7.0), the resulting conformations are not equivalent to those normally found in the native tissue [28]. Consequently, hydrogels formulated solely from dECMs often exhibit poor architectural characteristics and mechanical stability [17,28,29], which translates into suboptimal tissue maturation processes. To overcome these impediments, a variety of external stimuli have been explored to improve the performance of dECM hydrogels. Several physical stimuli (e.g., temperature, light, strain, stress, electric, magnetic, and acoustic) have been implemented to improve the mechanical stability of dECM-based hydrogels through alternative crosslinking mechanisms or by tuning their microarchitecture according to specific tissue hallmarks. The microarchitecture is, in fact, one of the most important performance parameters when these hydrogels are intended for the engineering of physiologically relevant tissues [30,31]. Moreover, considering that the functionality of many native tissues is dependent on built-in mechanisms that promote dynamic behaviors in response to external stimuli (e.g., muscle contraction, neuronal electrical transduction, and articular loading), construct stimulation during maturation is thought to help guide the development of tissues with functional phenotypes [32]. On this matter, several stimuli-responsive materials and biomolecules have been combined with dECM-based hydrogels to guide their response to external stimuli. This approach has also enabled numerous applications in the localized delivery of therapeutics, considering that the release of loaded pharmacological agents can be precisely controlled through finely tuned responses [33].

In this review, we outline recent advances in the development of dECM-based hydrogels, understood as those derived from whole ECMs or based on their major components (e.g., collagen, HA, and fibronectin) for TE and RM applications. We particularly focus on elucidating how different types of external stimuli have been employed for improving their performance, both for cellular scaffolding and for the controlled delivery of therapeutics (summarized in Table 1). Our aim is to highlight the protagonism of biomimetic materials with tunable stimuli-responsive properties on recent advances in biofabrication and drug delivery, as well as to address their potential for future developments in these fields.

## 2. Enhancing the Mechanical Properties of dECM-Based Hydrogels

**Figure 1:**
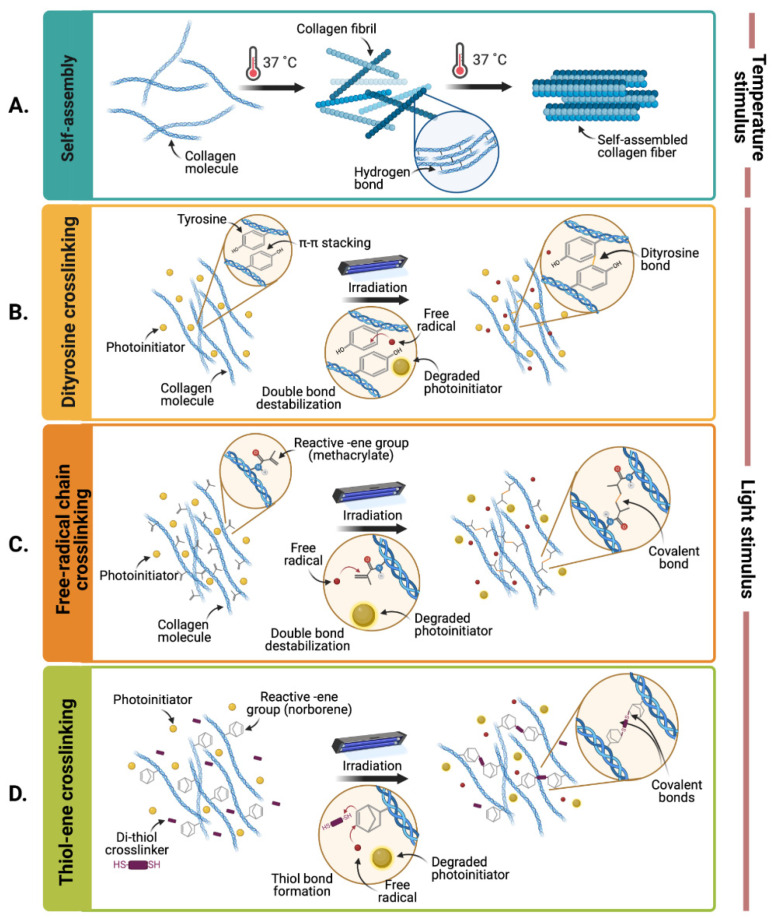
Hydrogel crosslinking upon thermal and light stimuli. (**A**) Self-assembly of collagen molecules upon thermal stimulus. (**B**) Photo-induced crosslinking between tyrosine residues, (**C**) methacryloyl, or (**D**) norbornene moieties (Created with BioRender.com).

As the main component of the majority of ECMs is type I collagen, the most straightforward approach for inducing gelation of dECM-derived hydrogels is through thermal stimuli [60]. Type I collagen molecules are triple-helical structures that, under physiological conditions of temperature and pH, are able to assemble into hierarchically organized structures that interact through hydrogen bonding forces (Figure 1A) [61,62]. In native tissues, collagen molecules assemble into fibrillar structures, which, in turn, organize into fibers and create the characteristic fibrous backbone of ECMs. However, collagen crosslinking dynamics are not fully preserved on ECM-derived hydrogels, mainly as the decellularization and enzymatic solubilization processes to produce them largely disrupt their organizational hierarchy and therefore compromise their ability to reassemble [21,63]. Moreover, thermal crosslinking is considerably limited by the heat transport rate within the surrounding environment, which results in slow gelation processes that, in turn, lead to insufficient structural stability over time, as evidenced by reversible sol–gel transitions [64]. For this reason, when extruded through 3D bioprinting devices, thermally crosslinked dECM hydrogels normally exhibit poor shape fidelity [65]. This is concerning from the tissue engineering viewpoint, as the degradation kinetics of dECM-based hydrogels are usually faster than the required matrix remodeling rates for the timely maturation of biofabricated constructs and for in vivo regeneration [62,66]. Accordingly, there is a critical need to improve the biomechanical performance of dECM-based hydrogels such that they can be successfully implemented in tissue engineering applications with the ultimate goal of clinical translation. In order to address this limitation, several methods have been employed, which we discuss in detail below [61,65,67].

### 2.1. Improving Thermal Gelation Dynamics

With the purpose of reducing the gelation time of a porcine skin dECM bioink upon deposition, a 3D cell printing system equipped with two heating modules located above and below the construct was devised by Anne and colleagues [68]. They found that this setup, combined with an increase in the dECM concentration of the hydrogels, improved the structural stability of dECM constructs over time. This was due to the faster gelation kinetics triggered by the setup and the denser hydrogel networks, which facilitated the self-assembly of ECM proteins into tissue-like patterns. However, although a 2.5% (*w*/*v*) concentration presented the best mechanical performance, increasing hydrogel viscosity inadvertently reduced cell viability, which hampered the possibility of fabricating more stable constructs [68]. Alternatively, several researchers have incorporated polymeric frameworks during dECM-based hydrogel deposition to add structural support without the need of high dECM concentrations [18,61,69]. For instance, Pati and colleagues bioprinted adipose tissue and cartilage-derived dECM bioinks on a polycaprolactone (PCL) framework that provided sufficient structural support to allow adequate thermal gelation and tissue maturation without significantly reducing the viability of embedded human adipose tissue-derived stem cells (hASCs) and human inferior turbinate-derived stromal cells (hTMSCs) [70]. Their bioprinting process consisted of the sequential layer-by-layer deposition of the PCL frame and of the dECM bioink in every alternating gap between PCL filaments. By acting as an external structure that supported the constructs, the PCL helped to relieve the low mechanical properties of the dECM bioink before thermal crosslinking and during construct maturation. Likewise, Lee and colleagues bioprinted a liver dECM hydrogel embedded with human bone marrow derived mesenchymal stem cells (hBMMSCs) within a PCL framework in a similar fashion [71]. In both studies, the use of a frame granted a structurally stable and biocompatible microenvironment for the proliferation and differentiation of embedded human stem cells into tissue-specific lineages.

### 2.2. Photocrosslinking

Stimulation with light has been an alternative strategy for strengthening the mechanical properties of hydrogels, as it can initiate photocrosslinking reactions in a wide variety of biomaterials [72,73,74]. These reactions typically occur in the presence of a photoinitiator molecule (e.g., riboflavin (RF), riboflavin phosphate (RFP), lithium phenyl-2,4,6-trimethylbenzoylphosphinate (LAP), 2-hydroxy-4′-(2-hydroxyethoxy)-2-methylpropiophenone (Irgacure 2559), ruthenium/sodium persulfate (Ru/SPS)), which forms reactive radical species upon light-mediated degradation [67,74]. Once generated, these species can react with specific functional groups on the main backbone chain of ECM proteins to form covalent bonds or reactive radical intermediates [74]. Ultraviolet (UV) light (100–400 nm), blue light (400–490 nm), and white light (400–700 nm) are the most common light stimuli for inducing photocrosslinking reactions. However, as UV light is known to induce DNA mutations and cell death, it is the least preferred wavelength range for photocrosslinking reaction schemes [35,75].

Notably, dECM hydrogels have shown photosensitive properties in the presence of photoinitiators due to the destabilization of nearby collagen or proteoglycan residues. Although the precise mechanisms of these reactions are elusive, it has been reported that tyrosine and histidine residues may be involved in this light-induced crosslinking approach [76,77,78]. This is as tyrosine residues form π–π complexes that facilitate dityrosine bonds in the presence of singlet oxygen (Figure 1B) [76], and the imidazole group of histidine residues is easily oxidized to transient intermediates that can lead to new crosslinked products [77].

Numerous studies exploring the photocrosslinking dynamics of collagen hydrogels in the presence of RF [79,80,81,82], have demonstrated a consistent increase in elastic and compressive moduli upon blue-light [81] or UV [79] exposure. Similarly, our research group developed a photoresponsive hydrogel by mixing small intestinal submucosa (SIS) dECM with RF that photocrosslinks in response to blue light. We reported that an increase in RF (up to 0.5% (*w*/*v*)) led to a proportional increase in the storage modulus from 2 kPa to 4 kPa [75]. Jang and colleagues developed a heart dECM hydrogel, also with RF, which demonstrated increased mechanical properties upon UVA light exposure. Additionally, the material exhibited an entropy-driven self-assembly of collagen with an increase in temperature to 37 °C [66]. With this dual crosslinking scheme, the compressive modulus of these hydrogels increased from 0.18 kPa to 15.74 kPa, and the dynamic complex modulus from 0.33 kPa to 10.58 kPa.

Recently, Kim and colleagues showed that using Ru/SPS as a photoinitiator system potentiated dityrosine crosslinking in corneal and heart dECMs upon blue-light exposure [41]. Due to its high absorptivity within this light range (400–450 nm), high molar extinction coefficient, and high chemical stability in its excited state, Ru/SPS allowed efficient curing reactions at relatively low concentrations (0.5–2 mM) and exposure time (5 s), which demonstrates better crosslinking dynamics than RF. In particular, they show that Ru/SPS-mediated dityrosine crosslinking increased the compressive modulus of corneal and heart dECMs up to 60 and 70 kPa, respectively. However, their complex modulus remained below 1 and 0.5 kPa. Moreover, with extrusion-based and digital-light processing bioprinting technologies, they built multi-layered and complex geometries with both bioinks and demonstrated that their method allows the additive manufacturing of constructs with high shape fidelity.

However, beyond the utilized photoinitiator, the efficiency of these photocrosslinking reactions is highly limited by the low concentration of reactive tyrosine or histidine residues on dECM protein backbones [78]. Therefore, recent efforts have focused on increasing the number of photosensitive groups that can participate in these crosslinking reactions.

#### Biochemically Modified dECM-Based Hydrogels with Augmented Photosensitivity

Several research groups have potentiated photocrosslinking in dECM-based hydrogels by the addition of biochemically modified natural polymers with enhanced photosensitivity. These biochemical modifications introduce pendant groups with reactive double bonds (e.g., methacrylate, acrylate, norbornene, vinyl ethers, and N-vinyl amide) [74] that can be easily destabilized in the presence of free radicals, thus facilitating the formation of covalent bonds and increasing the efficiency of photocrosslinking (Figure 1C,D). Methacryloyl-modified gelatin (GelMA) is perhaps the most commonly implemented biochemically modified material due to its high biocompatibility, ease of processing and rapid crosslinking upon light exposure [18,83]. Bejleri and colleagues, for example, developed a photoresponsive GelMA (0.1 mM)-based bioink mixed with cardiac dECM and eosin Y as a photoinitiator (5% (*w*/*v*)) for bioprinting cardiac patches [35]. This crosslinking scheme yielded a homogeneous distribution of dense fibers within the bioprinted constructs without reducing cell viability. The incorporation of GelMA was reported to significantly increase the storage modulus of the bioprinted constructs after the photocrosslinking process. Furthermore, Skardal and colleagues developed a porcine liver dECM-based bioink mixed with thiolated HA and thiolated gelatin, two different PEG-based crosslinkers (PEG-acrylate and PEG-alkyne) and a photoinitiator (Irgacure 2959) [34]. This bioink spontaneously crosslinked at neutral pH due to the formation of thiol-acrylate bonds between the PEG-acrylate and the thiolated polymers. However, further crosslinking was achieved upon pendant thiol group exposure to UV light, due to the photo-induced near-instantaneous thiol-alkyne polymerization reaction with the PEG-alkyne crosslinkers. This study also reported that the stiffness increased by nearly 200-fold (i.e., from 0.1 to 19.8 kPa) by varying the concentration, molecular weight, and geometry of the alkyne-modified PEG crosslinker.

Direct biochemical modifications to dECM proteins have also become recurrent approaches as they eliminate the need of incorporating additional materials. For instance, thiol- and methacryloyl-modified HA hydrogels were investigated by Lee and colleagues for developing prolonged-action delivery vehicles to the ocular surface [36]. The modification of HA with these pendant groups, in combination with the addition of 0.01% (*w*/*v*) RF as photoinitiator, allowed forming dense photocrosslinked networks upon blue light exposure, which can be attributed to thiol–ene reactions between methacryloyl and thiol groups. They demonstrated that the photocrosslinking process prolonged the stability of the hydrogel, and dictated the release of loaded bovine serum albumin (BSA). Wu and colleagues also developed a photocrosslinkable construct to fabricate personalized pharmaceutical tablets with controlled dosages of active pharmaceutical ingredients, both hydrophilic and hydrophobic (e.g., lisinopril and spironolactone) [84]. The manufactured constructs consisted of two layers. First, a lisinopril-loaded hydrophilic hydrogel based on 3% (*w*/*w*) norbornene-functionalized HA mixed with PEG dithiol (PEGDT) supplemented with 10% (*v*/*v*) eosin Y as photoinitiator and 10% (*v*/*v*) PEG. Second, a spironolactone-loaded hydrophobic hydrogel based on 30% (*w*/*w*) PEG diacrylate (PEGDA), 50% (*w*/*w*) PEG, and 20% (*v*/*v*) ethanol supplemented with both 1 mM eosin Y as photoinitiator and 0.05 M mPEG-amine as co-initiator. The layers were dually photocrosslinked upon visible light exposure due to thiol–ene reactions between thiol groups of PEGDT and norbornene groups of HA, and the covalent bonds formed between destabilized acrylate groups of PEGDA, respectively. They demonstrated that, irrespective of drug loading concentration, but with the appropriate light dosage, these tablets achieved optimal mechanical properties and dual sustained release profiles over time [84].

**Figure 2:**
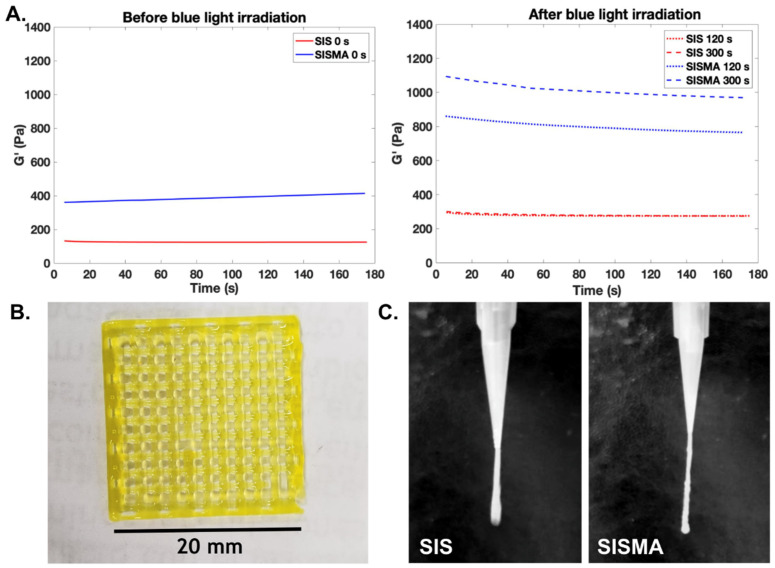
(**A**) Methacryloyl-modified small intestine submucosa dECM (SISMA) hydrogel bioprinted into a square lattice with high shape fidelity. (**B**) Filament formation of unmodified SIS and SISMA hydrogels made at the same ECM concentration (25 mg/mL) upon extrusion as a key feature for adequate printability. (**C**) Storage modulus of unmodified-SIS and SISMA hydrogels before and after photocrosslinking with varying exposure time. SISMA hydrogels exhibit superior mechanical stability, before and after blue light irradiation, and superior crosslinking dynamics.

Direct biochemical modifications to dECM hydrogels have also been conducted. For instance, Ali and colleagues successfully bioprinted a bioink based on methacryloyl-modified kidney dECM (KdECMMA) and mixed with HA, glycerol, and gelatin [38]. The storage modulus of irradiated KdECMMA constructs increased 1.7-fold with respect to constructs fabricated with unmodified kidney dECM (KdECM). Moreover, the stiffness of KdECMMA hydrogels increased proportionally to dECM concentration, but remained unaltered at a low level for KdECM hydrogels. Consequently, KdECMMA hydrogels presented a lower degradation rate and helped maintain the stability of entrapped bioactive molecules by protecting them from early enzymatic degradation. Similarly, our research group developed methacryloyl-modified SIS dECM hydrogels (SISMA) mixed with 0.05% (*w*/*v*) RF, which significantly potentiated their mechanical properties upon blue-light irradiation without the need for excipient materials. As shown in Figure 2A, the storage modulus of SISMA hydrogels is around 3 and 3.5 times greater than that of unmodified hydrogels before and after blue light exposure for 5 min. Furthermore, the photocrosslinking reaction in unmodified hydrogels saturates rapidly, yielding a similar storage modulus after 2 min and 5 min of exposure, while that of SISMA hydrogels increases according to exposure time. These results support the notion that this biochemical functionalization significantly improves the overall mechanical stability of hydrogels and their crosslinking dynamics, resulting in superior printability and enhanced shape fidelity of constructs (Figure 2B,C).

Similarly, Parthiban and colleagues developed a methacryloyl-modified bone dECM hydrogel (BoneMA) mixed with 0.15% (*w*/*v*) LAP which, upon photocrosslinking, supported the formation of interconnected vascular networks from embedded human umbilical vein endothelial cells (HUVECs) [39]. The elastic modulus of photocrosslinked BoneMA hydrogels also increased according to exposure time, which demonstrates that this crosslinking method allows highly tunable mechanical properties [85]. BoneMA constructs showed a higher total vessel length and visibly faster network formation than GelMA constructs (which have shown vasculogenic potential), possibly due to the retention of pro-angiogenic growth factors from the tissue of origin, which promoted faster vascularization in vitro [39].

## 3. Tunning Microarchitectural Characteristics of dECM-Based Hydrogels

Although the composition of dECM-based hydrogels is a major contributor to their superior bioactivity, biomimicry is not only important composition-wise, but also architecture-wise [86]. The structural organization of ECM components in vivo imparts highly specialized biomechanical characteristics that are specific to tissue location and function. For instance, a linear orientation of ECM fibrillar components predominates within tissues exposed to high tensile stresses (e.g., tendons and some ligaments), whereas circumferential organization predominates within tissues that experience multiaxial tension, compression, and shear (e.g., annulus fibrosus and meniscus) [87]. The spatial distribution of cells within tissues is a major contributor to the microstructure as well, considering that organized cellular interactions are often necessary for specific tissue functions. This is typically observed within muscular tissues, as muscle cells must hierarchically organize into myotubular structures and then muscle fibers to ensure cooperative interactions during contraction [88]. The precise structural organization of hydrogel microenvironments upon deposition is therefore an essential feature for developing constructs with tissue-specific functionalities. Controlling the initial structural properties and distribution of embedded cells can dictate cellular fate (e.g., from pluripotency to specific differentiation profiles) and posterior matrix remodeling in uniquely prepared structural patterns.

Although the controlled patterning of hydrogels is highly effective in recapitulating the macro-geometry of tissues, it often fails in replicating micro-architectural characteristics. A wide variety of tissue architectures have been induced in dECM-based hydrogels through several types of stimuli including mechanical, magnetic, electric, and acoustic. These approaches are discussed below.

### 3.1. Mechanical Stimuli

**Figure 3:**
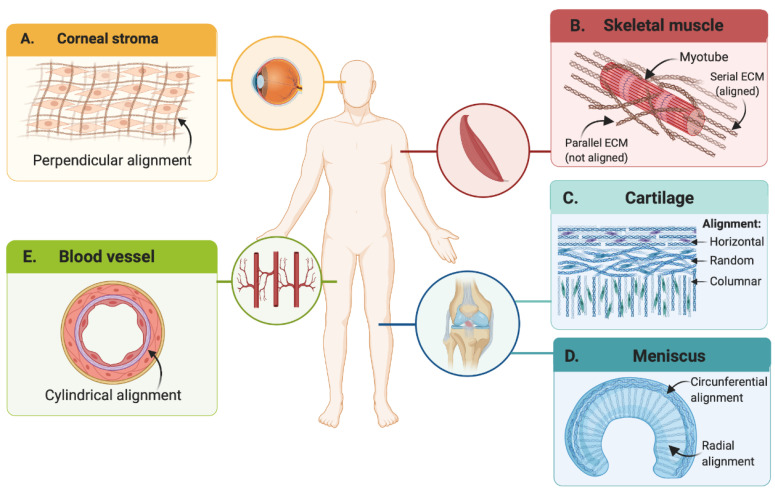
Examples of hierarchical cellular organization and ECM architecture in different native tissues. (**A**) Corneal stroma, (**B**) skeletal tissue, (**C**) cartilage, (**D**) meniscus, and (**E**) blood vessels (Created with BioRender.com).

As many tissue architectures require the anisotropic alignment of fibrous ECM proteins (mainly collagen) [86], the most straightforward approach has been to tune the shear stress during extrusion to induce their alignment in the direction of flow. This mechanical stimulus was exploited by Kim and colleagues for the unidirectional alignment of the collagen fibrils of a corneal stroma-derived dECM (Co-dECM) bioink to reproduce the architecture of native corneal stroma after tissue maturation [89]. They found that by increasing the nozzle gauge up to 25 G it was possible to induce collagen fibril alignment and create anisotropic structures that guide keratocyte orientation into linear patterns. This initial cellular distribution led to the secretion of collagen fibrils during keratocyte maturation in perpendicular directions, which, in turn, yielded the characteristic crisscrossed pattern of native corneal stroma that is fundamental for corneal transparency (see Figure 3A) [90]. The shear-induced alignment of collagen fibrils was also performed by Schwab and colleagues to reproduce the architecture of knee articular cartilage [91], which comprises three distinct layers: a superficial layer with fibrils tangentially oriented to the joint surface, a middle layer with random orientation, and an internal layer with columnar alignment (see Figure 3C) [86]. With a computer-aided design (CAD), they printed a tyramine-HA (THA)-collagen I bioink with embedded human mesenchymal stem cell (hMSC) spheroids. The superficial layer was printed in horizontal filaments while the internal layers were formed by circular-vertical structures. As expected, the initial shear-induced alignment of collagen fibrils guided the unidirectional migration of hMSCs from spheroid micropellets and significantly increased cytoskeleton alignment along the direction of fibrils, which was not observed in isotropic THA-collagen I hydrogels. Recently, another research group reported an alternative method to increase the shear-induced cellular alignment in bioprinted constructs by pre-incubating methacryloyl-modified collagen (ColMA) bioinks with myoblasts (C2C12) [92]. Their rationale was that the pre-incubation period promoted cell–matrix interactions before printing, which induced cell transition from spherical to elongated morphology and favored their alignment during extrusion. Moreover, according to their findings, pre-incubated constructs had significantly higher cytoskeleton alignment and superior myotube formation after 21 days, when compared with constructs that were not pre-incubated.

In addition to shear-stress, other mechanical stimuli, such as strain, have been explored for the microarchitectural remodeling of bioprinted constructs [93,94]. For instance, Puetzer and colleagues induced circumferential alignment of collagen fibers within a meniscus-replicating construct [95]. They anchored the meniscus model at the horns to mimic the native tibial attachment sites in vivo, and to restrict the compaction of the collagen hydrogel, a phenomenon commonly observed during the maturation of dECM-based constructs. This geometrical constraint induced residual hoop stress circumferentially and encouraged the alignment of fibrils. Clamped menisci developed native-like sized and circumferential fibers in the outermost surface, as well as radially aligned fibers in the innermost surface, thereby resembling native tissue after 8 weeks of culture (see Figure 3D). This approach was also explored by Choi and colleagues for the development of anisotropically aligned skeletal muscle constructs. Accordingly, they bioprinted a skeletal muscle-derived dECM bioink with embedded myoblasts (C2C12) in a PCL anchoring set up that geometrically constrained opposite ends of the construct. This generated a longitudinal constant strain by opposing hydrogel compaction during the maturation process [94]. With this mechanical stimulus, they reported that 76% of cells were unidirectionally aligned after 7 days and with an elastic modulus comparable to that of native skeletal muscle within 14 days of incubation. Most importantly, striated band patterns were observed within myotubular structures, which indicated that the contractile apparatus of native muscle was successfully formed, implying structural and functional maturity.

### 3.2. Electrical Stimuli

Other approaches have coupled mechanical with electrical stimulation for the engineering of hierarchically organized and functional muscle tissue, considering that beyond mechanical response it also displays inherent electrosensitive properties. In addition to anisotropic cellular alignment, mature muscular phenotypes have specific ECM patterns around myotubular structures to allow adequate muscle contraction. Collagen IV fibers, which attach directly to sarcolemmas and connect them to other types of collagen [96], exhibit a lower degree of alignment/stiffness in parallel locations to myotubes to minimally interfere with myotube contraction. In contrast, fibers located in series with myotubes have a higher degree of alignment to bear and transmit the contraction forces to the imposed load (see Figure 3B) [97]. Accordingly, Kim and colleagues demonstrated that by applying an out-of-phase mechanical and electrical co-stimulation scheme (for short exposure periods of 20 min) to myoblast-laden Matrigel/fibrinogen constructs previously matured to skeletal muscle tissue, they remodeled into the characteristic muscular structural patterns [98]. The rationale behind this approach is that mechanical stretching generates tensile stress along the load direction, while muscle contraction triggered by an electric potential induces shear and compressive stress on collagen IV fibers parallel to myotubes and tensile stress at intrafascicularly terminating ends [98]. They showed that an alternating application of these two stimuli yielded a superior contraction force in the matured constructs compared to applying either of the two stimuli separately or simultaneously. The synergistic stimulation scheme effectively maximized fiber alignment in serial ECM locations and minimized it in parallel ECM locations.

Electrical stimulation alone, for longer time periods, has also been employed for controlling cell alignment within ECM-based hydrogels [99]. Electrosensitive cell types (e.g., cardiomyocytes, myoblasts, and neural stem cells) align parallel to the electric field vector to maximize the field gradient across them, whereas other types of cells (e.g., adipose-derived stromal cells, or endothelial progenitor cells) align perpendicularly to minimize it [100]. However, the effectiveness of electrically induced cellular alignment has proven to be low and to require long stimulation periods that, ultimately, may reduce cell viability [101]. The incorporation of rod-shaped electrosensitive nanostructures (e.g., carbon nanotubes (CNTs) and gold nanowires (GNWs)) into ECM-based hydrogels has emerged as a plausible alternative to guide their electrical response as they can easily align with the direction of the applied electric field and therefore facilitate the alignment of surrounding microstructures [51,102]. Kim and colleagues, for example, embedded GNWs in a myoblast-laden collagen bioink and demonstrated that the electrically induced alignment of these nanostructures significantly narrowed the fiber orientation distribution. This, in turn, favored myogenic differentiation compared with electro-stimulated myoblast-laden collagen constructs in the absence of GNWs and non-electrostimulated constructs [51].

### 3.3. Magnetic Stimuli

The incorporation of magnetic nanostructures, specifically iron oxide nanoparticles (IONs), has also shown great promise for the alignment of ECM fibers due to their high sensitivity to externally applied magnetic fields. A recent work by Betsch and colleagues showed that streptavidin-coated IONs incorporated within collagen–agarose hydrogels successfully guided the anisotropic alignment of collagen fibers for the recreation of the cartilage microarchitecture [54]. Upon exposure to an external magnetic field during the temperature gelation process, collagen fibers were forced to align unidirectionally due to the traveling motion of these nanoparticles across the hydrogel. The authors showed that collagen fiber alignment in all formulated hydrogels more than doubled their compressive tangent modulus and yielded mechanical features that were similar to those of the native cartilage. Moreover, the fabrication of a two-layered construct, consisting of an anisotropically aligned superficial layer and a randomly oriented internal layer led to remarkable collagen I and II secretion by embedded primary knee articular cartilage cells than in any of the bioprinted samples of either of the two individual layers. This suggests that the combined microarchitectural characteristics of the two-layered construct, which is similar to native cartilage, significantly improves chondrocyte maturation. Similarly, Rose and colleagues developed a magnetically responsive hybrid hydrogel consisting of (i) rod-shaped acrylate-modified poly(ethylene oxide-*stat*-propylene oxide) microgels loaded with monodispersed IONs in (ii) a surrounding fibrin (i.e., polymerized fibrinogen) matrix with embedded chicken-derived primary dorsal root ganglions (DRGs) [55]. Upon exposure to a low-intensity magnetic field (in the mT order), the rod-shaped microgels aligned well along the field direction. Their orientation was subsequently fixed with an in situ fibrin crosslinking treatment. Although embedded microgels fail to provide cell adhesion focal points and fibrin fibers are not directly aligned, microgel alignment was sufficient to guide neurite growth parallel to their direction. This novel approach demonstrated that minimal structural guidance can trigger nerves to grow unidirectionally.

### 3.4. Acoustic Stimuli

The development of complex microarchitectures with non-linear patterns within ECM-based hydrogels is an ongoing challenge due to the requirements in terms of means for high spatial control. In this regard, acoustophoretic systems have shown the most promising results as demonstrated by the guided migration of cells into specific patterns that follow the pressure fields formed by standing surface and standing bulk acoustic waves [103,104]. In addition to favoring the development of hydrogel constructs with high cellular alignment, acoustophoresis has been used to organize embedded cells within hydrogels into the cylindrical structures needed to guide the fabrication of vascular networks. As reported by Kang and colleagues, HUVECs and human adipose-derived stem cells (hADSCs) embedded within a catechol-functionalized HA hydrogel were successfully co-aligned into collateral cylindrical patterns (see Figure 3E) [105]. This was enabled by employing surface acoustic waves of 280 µm wavelength, which concentrated the cylindrical patterns at intervals similar to the intercapillary distance of human skeletal muscle. This initial cell patterning led to both interconnected capillary networks due to the enhanced branching between parallel structures, and the organized formation of functional endothelial barriers. Moreover, these mature constructs were able to successfully integrate with host vasculature after subcutaneous transplantation into dorsal regions of mice, forming perfusable and interconnected networks with native microvessels. This was in contrast with constructs that were not patterned.

Similarly, Petta and colleagues proposed an acoustic-based technology, termed sound induced morphogenesis (SIM), in which they combine acoustic patterning with physiological self-assembly to generate multi-scale and perfusable vascular networks [106]. With vertical mechanical vibrations (80 Hz and 0.5 g acceleration amplitude), they generated acoustic surface standing waves that patterned HUVEC and hMSC spheroids in concentric rings within a fibrin hydrogel. They showed that the small distance between adjacent rings favored HUVEC sprouting from the patterned spheroids in capillary-like structures that fused with sprouts from adjacent rings, thus creating microvessel-like morphologies. Moreover, when embedding an endothelialized macrochannel through the center of the hydrogel, simulating a macrovessel, the capillary-like sprouts from patterned spheroids were able to fuse with this structure as well, and create perfusable multiscale vascular networks after only 5 days.

Despite these efforts on recreating vascular networks, these works employ simple and symmetric geometries that may not recapitulate the arbitrary and non-symmetrical arrangements of vasculature in most biological tissues. To circumvent this, acoustic holography was recently proposed by Ma and colleagues for constructing sophisticated 3D cell patterns according to complex pressure fields [107]. In this technique, complex patterning is enabled by propagating a 5 MHz acoustic plane through a 3D-printed topography (termed the ‘hologram’). This is precisely designed to induce a specific phase field, which is then projected towards the hydrogel to induce cell arrangement according to the generated pressure field. Accordingly, by changing the topography of the hologram, it is possible to obtain desired patterns on demand. As a proof-of-concept, they demonstrated that human colon cancer cells (HCT-116) embedded in collagen hydrogels could be patterned with this technique into complex structures (e.g., asymmetric geometric patterns, human profiles), prior to temperature gelation and without significant impact on cell viability. Although acoustic holography is yet to be employed for the development of physiologically relevant structures, their work demonstrated the potential of this technology in tissue engineering and mechanobiology.

## 4. Improving Morphogenesis and Functionality of dECM-Based 3D Cultures with External Stimuli

Although the mechanical and micro-architectural characteristics of dECM-based hydrogels significantly influence cell behavior [108,109], in most cases, they are not enough for ensuring full functionality in vitro. This is as they fail to replicate the myriad of biochemical signals originating from the native extracellular environment, which are essential for directing cellular fate [110]. During maturation, cells must be able to communicate with each other and their niche through biochemical and mechanotransductional cues, as these interactions induce morphogenetic signals that ultimately lead to tissue functionality [111]. Therefore, ensuring an adequate maturation process is key for directing morphogenesis and the hierarchical organization of tissues at the macro and micro scales. Several strategies that employ external stimuli for directing specific maturation profiles in cell laden dECM-derived hydrogels are discussed below.

### 4.1. Directing Stem Cell Differentiation

**Figure 4:**
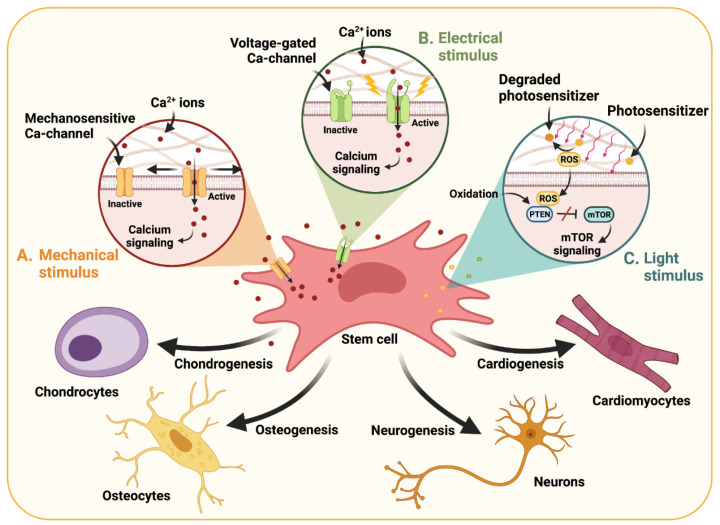
Schematic of the modulating effect of (**A**) mechanical, (**B**) electrical, and (**C**) light stimuli on the activation of specific signaling pathways that direct differentiation profiles of stem cells. Specific stimulation regimes that mimic mechanotransductional or electrical cues in embryonic development can activate calcium signaling. Similarly, ROS production upon light stimulation can promote the oxidation of PTEN, a strong inhibitor of mTOR, and thus upregulate mTOR signaling [121]. (Created with BioRender.com).

One of the most valuable applications of external stimuli in TE and RM is directing stem cell differentiation into specialized and adult-like phenotypes [112]. Mechanical and electrical stimulation, for example, have been widely explored for directing the differentiation of human mesenchymal stem cells (hMSCs) towards osteochondral lineages [113]. This has been attributed to the modulating effect of such stimuli over calcium (Ca^2+^) influx towards intracellular compartments. In this regard, specific intracellular Ca^2+^ profiles have shown to direct chondrogenesis and osteogenesis in vivo, as calcium channels participate in the transduction of mechanical signals that direct these differentiation profiles during embryonic development [114,115]. Notably, brief and pulsed Ca^2+^ influx periods during early differentiation stages have shown to promote MSC chondrogenesis, whereas prolonged Ca^2+^ exposure periods promote osteogenesis [116,117]. Accordingly, these physiological hallmarks have been harnessed to promote the osteochondral differentiation of MSC-laden dECM-derived hydrogels by modulating the activity of these channels through mechanical stimuli [118]. Alternatively, it is possible to control the activity of voltage-gated calcium channels with electrical stimuli (Figure 4A,B) [116,119,120].

For instance, Aisenbrey and colleagues studied the chondrogenic effect of mechanical stimuli on cartilage mimetic hydrogels with embedded human induced pluripotent stem cell (hiPSC)-derived mesenchymal progenitor cells (MP-iPSCs) [122]. Their mimetic hydrogels consisted of 8-arm PEG functionalized with norbornene, PEG dithiol crosslinkers, and thiolated chondroitin sulfate, which is the main proteoglycan of cartilaginous tissues. The obtained cylinder-like constructs were subjected to mechanical compressive strains daily, during 1 h for 3 weeks, and the combined effect of this dynamic culturing with the presence or absence of differentiating growth factors was explored (e.g., TGFβ2 and BMP2). Their results showed that the mimetic hydrogel alone induced morphogenetic signatures, as evidenced by chondrogenic gene expression and immunohistochemical analyses. However, the combination of dynamic culturing and growth factor supplementation synergistically induced chondrogenesis while limiting tissue hypertrophy, which has been a main drawback observed on static culturing in the absence of growth factors. Similarly, Choi and colleagues showed that simultaneous growth factor supplementation and mechanical stimulation with low-intensity ultrasound (10 min/day, 1 MHz) also promoted chondrogenic differentiation in MSC-embedded fibrin-HA hydrogels. After 28 days, an increased sulfated GAG and collagen synthesis was observed in the stimulated constructs, which is a characteristic behavior of mature chondrocytes [118].

Regarding electrical stimuli, Vaca-Gonzalez and colleagues recently showed that intermittent 60 kHz electrical stimulation of hMSCs-embedded HA-gelatin hydrogels (30 min, 4 times per day for 21 days) not only enhanced GAG and type II collagen synthesis, but significantly increased the expression of chondrogenic markers (e.g., SOX-9 and aggrecan) without the need for differentiating growth factors [123]. Moreover, longer electrical stimulation periods of 4 h performed 3 times/day (7.6 mV/cm, 10 Hz) on type I collagen- and HA-coated PCL scaffolds, seeded with MSCs, significantly promoted osteogenesis [124]. This was demonstrated, after 28 days of stimulation, by the increased alkaline phosphatase (ALP) activity and increased expression of osteogenic markers (e.g., osteopontin, osteocalcin, and ALP).

The modulation of Ca^2+^ signaling has also been harnessed for the differentiation of other types of stem cells towards electrically active lineages, such as neural, cardiac, or skeletal muscle cells [125,126]. In this case, the increased Ca^2+^ handling induced by external stimuli has shown to promote early electrical/contractile activity by upregulating expression profiles of genes related to voltage-gated ion channels, which ultimately directs their maturation towards electrically active phenotypes [127]. However, as these differentiation routes require higher intensity and sustained electrical currents, most approaches have employed nanocomposite hydrogels embedded with electroconductive nanostructured materials to achieve superior electrical percolation networks within the hydrogel. This, in turn, ensures an early electrical connectivity within embedded cells, and facilitates their differentiation towards electrically active lineages. In this regard, carbon-based nanostructures such as graphene, reduced graphene oxide (rGO) [46,128], and carbon nanotubes (CNTs) [47,129], have been widely explored for their exceptional electroconductivity [130]. Nonetheless, a major setback in their implementation has been their limited cytocompatibility and poor dispersibility in hydrophilic media, which result from their inert sp^2^ hybridized carbon backbone [131].

To circumvent this, several studies have functionalized their surfaces with hydrophilic agents [48,132,133], controlled the reduction degree of graphene oxide (GO) by using weaker reducing agents [49], or included hydrophobic moieties within the hydrogels to promote hydrophobic or π–π stacking interactions with these nanostructured materials [129]. For instance, Shin and colleagues devised a catechol-functionalized HA hydrogel where CNTs were stably dispersed along with polypyrrole nanocomposites through hydrophobic interactions with the present catechol moieties [129]. They showed that these electroconductive motifs significantly promoted the differentiation of hiPSC-derived neural progenitor cells and human fetal neural stem cells with improved electrophysiological functionality, as demonstrated by the upregulation of calcium channel expression and improved depolarization dynamics. Alternatively, our group proposed a biocompatible, in situ reduction scheme for GO embedded within SISMA bioinks, which harnessed the enhanced dispersibility and bioactive properties of GO at initial maturation stages, and subsequently increased its electroconductivity by two orders of magnitude upon partial reduction aided by ascorbic acid exposure [40]. With this maturation scheme, embedded human adipose-derived MSCs maintained high viability levels, as well as enhanced cell adhesion and proliferation, which demonstrated the enormous potential of this scaffold and maturation scheme for facilitating biocompatible electrical stimulation regimes that can potentially guide their differentiation towards neurogenic or myogenic phenotypes. Carboxyl-functionalization of CNTs was also proposed by Ahadian and colleagues to facilitate their stable dispersion in GelMA hydrogels [47]. Their incorporation significantly enhanced the differentiation of mouse embryonic bodies into cardiogenic phenotypes, as demonstrated by the upregulation of cardiogenic markers (e.g., Tnnt2, Nkx2-5, and Actc1) and increased contractile function upon electrical pulse stimulation of 3V at 1 Hz for 2 days. Although GelMA hydrogels are not strictly ECM-derived, this dispersion method can be easily extrapolated to ECM-derived hydrogels considering that gelatin is a hydrolyzed form of collagen and, in turn, retains some of its physical and chemical properties [134].

The electrically guided differentiation of stem cells, through the incorporation of other electroconductive nanostructured materials, has also been studied with GelMA hydrogels. Black phosphorus (BP) nanosheets, for example, were explored by Xu and colleagues to enhance the neural differentiation of seeded MSCs [50]. They showed that the electrical stimulation (100 mV/cm) of BP-embedded GelMA hydrogels for a 7-day period significantly increased the expression of neuronal markers (e.g., nestin, Tuj1) when compared to electrostimulated and non-electrostimulated GelMA hydrogels. Heo and colleagues similarly showed that the potentiating effect of gold nanoparticles (GNPs) on hydrogel electroconductivity significantly improved osteogenic differentiation of MSCs. They demonstrated that GNP addition to MSC-embedded GelMA hydrogels induced a marked increase in osteogenic markers (e.g., BSP, OCN, COL1, and Runx2) and ALP activity [135]. The observed changes occurred in a dose-dependent manner after 14 days of incubation. These approaches exemplify other routes that could be considered for engineering electroconductive ECM-derived hydrogels that direct stem cell differentiation.

In addition to electrically or mechanically induced calcium signaling, intracellular ROS modulation has also shown to contribute to stem cell differentiation processes (Figure 4C) [121,136,137]. In this regard, light stimuli have been employed to induce ROS production by degrading embedded photosensitizers within the hydrogels. Lu and colleagues, for instance, demonstrated that the implementation of photodynamic therapy promoted the chondrogenic differentiation of bone marrow stem cells (BMSCs) embedded in type I collagen hydrogels conjugated with carbon dots through genipin crosslinkers [37]. The hydrogels were injected into a joint cartilage defect created on mice, followed by stimulation with a near-infrared (NIR) laser for 3 min every other day for 8 weeks. The stiffness of the crosslinked hydrogel and the non-lethal doses of intracellular ROS after stimulation synergistically upregulated cartilage-specific genes (e.g., SOX9, ACAN, and COL2A1) and enhanced GAG secretion. Moreover, the authors proved that increased ROS levels significantly promoted the activation of mTOR signaling, which is an important pathway in chondrogenic differentiation [138].

### 4.2. Maturation of Electrosensitive Tissues

Beyond its effect on directing differentiation profiles, electrical stimulation has also proved useful for achieving electrically mature phenotypes in differentiated myocardial and neural cells. The post-mitotic nature of terminally differentiated cardiomyocytes and neurons has hampered their use within 3D culture systems that need to be dynamically remodeled for proper functionality. Therefore, the use of phenotypically immature precursors has been the most straightforward and cost-effective solution, as it eliminates the need of acquiring pluripotent stem cells and implementing precisely controlled differentiation regimes every time [139]. Accordingly, several electroconductive dECM-based hydrogels have been envisioned for improving the maturation of these tissues by promoting electrically active phenotypes in biomimetic microenvironments [49]. Tsui and colleagues, for instance, developed an rGO-embedded myocardial dECM hydrogel that showed high hiPSC-derived cardiomyocyte viability after 35 days and increased contractile and electrophysiological function by tuning rGO reduction degree and concentration within the bioink [49]. Alternatively, Roshanbinfar and colleagues developed an electroconductive hydrogel based on pericardial tissue-derived dECM and carbodihydrazide-functionalized multi-walled CNTs (MWCNTs) with embedded hiPSC-derived cardiomyocytes for engineering cardiac tissue [140]. The embedded cells showed enhanced unidirectional orientation, greater contraction amplitude and speed, and improved calcium handling, when compared to the non-electroconductive counterparts. As a result, the presence of MWCNTs improved the beating properties of the constructs, as evidenced by the synchronous contraction of the engineered tissues without signs of arrhythmia.

Similarly, Koppes and colleagues developed a CNT-based electroconductive nanocomposite hydrogel but, instead of relying solely on the electric activity of the embedded cells, they explored the effect of a low-voltage direct current (DC) stimulation on the maturation of nerve tissue constructs [52]. They investigated this by manually casting tissue constructs from a Matrigel and type I collagen hydrogel embedded with carboxyl-functionalized single walled CNTs (SWCNTs) and dorsal root ganglia cells isolated from neonatal rats. The constructs were electrically stimulated for 8 h, incubated for 48 h, and subsequently fixed for studying neurite outgrowth. They found that the sole presence of the SWCNTs led to a 1.6-fold increase in the neural length and a 3.3-fold increase in the total outgrowth of cells, when compared to nanomaterial-free hydrogels. Moreover, relative to nanomaterial-free and non-stimulated tissue constructs, the electrical stimulation resulted in an exceptional 2.1-fold and 7-fold increase in neural length and total neurite outgrowth, respectively.

Interestingly, other studies have explored the effect of electrical stimuli on non-electroconductive hydrogels, and have shown that its coupling with mechanical stimulation compensates for the possible loss in electrical conductivity by reduced percolation. Ronaldson-Bouchard and colleagues developed a protocol for engineering mature human cardiac muscle based on the electromechanical stimulation of hiPSC-derived cardiomyocytes embedded on a fibrin hydrogel obtained after crosslinking of fibrinogen with thrombin [141]. With a custom-made bioreactor, newly formed cardiac tissue constructs were stimulated with a mechanical preload while applying electrical signals with the aid of two carbon rods placed in parallel to the tissue. Maturation was enhanced by slowly increasing the electrical stimulation frequency over the course of 2 weeks, which resulted in what the authors termed “intensity training”. This work, and the others mentioned previously, illustrate the importance of external stimuli during maturation stages for achieving superior morphogenesis in electrically active tissues.

### 4.3. Maturation of Load-Bearing Tissues

Mechanical stimuli have also been widely exploited for inciting mechanotransductional cues that guide functional phenotypes in load-bearing tissues. Typically, dynamic culturing of tissue constructs is employed to induce different types of stress on the materials, which can ultimately direct cell fate. Tensile and compressive forces, as well as fluid-induced shear stress, are among the most common stimuli for inducing such responses [142]. Goldfracht and colleagues explored the effect of dynamic culture conditions on cardiac tissue constructs fabricated with a composite hydrogel made of porcine heart dECM and chitosan [143]. Specifically, ring-shaped constructs were casted on molds, incubated for 24–48 h, and subsequently transferred to a passive stretcher device that exerted a constant tensile stress on them. Their results demonstrated enhanced morphogenesis as evidenced by continuous spontaneous contractions for up to six months. After 30 days under these dynamic conditions, embedded hiPSC-derived cardiomyocytes were arranged anisotropically, along the axis of stretch, and exhibited an organized sarcomeric pattern with distinguishable aligned Z bands. Additionally, the cardiomyocytes showed an upregulation in the expression of genes related to the contractile apparatus. Furthermore, the authors used programmed electrical stimulation to induce and map the development of arrhythmias, which demonstrated that the developed tissue models can be employed for in vitro disease modeling.

Similarly, the biosynthetic activity of primary human articular chondrocytes, embedded in methacryloyl-modified HA and GelMA (GelMA–HAMA) hydrogels, was shown to be significantly improved with the application of uniaxial and biaxial loads within precise mechanical stimulation regimes. As shown by Meinert and colleagues, uniaxial compressive and shear loads, applied independently and biaxially for 1 h after 14 days of 3D culturing, induced the immediate upregulation of hyaline cartilage-specific genes (e.g., ACAN, COL2A1, and PRG4) in an amplitude dependent manner [144]. Moreover, they show that intermittent biaxial loading applied at regular intervals (1 h daily at 1 Hz for 14 more days) promoted the secretion of type II collagen and the generation of a hyaline cartilage-like matrix in comparison to unstimulated constructs, without compromising cell viability. They suggest that these mechanical stimuli generate the necessary mechanotransductional cues for ensuring cartilage development and function. Likewise, Vasquez and colleagues showed that the mechanical loading of osteocytes controls osteoblast bone formation dynamics in a type I collagen 3D co-culture model [145]. In particular, they demonstrate that by applying a loading regime that consisted of cyclic compressions (2.5 N, 10 Hz, 5 min) to models pre-cultured for 7 days, prostaglandin E_2_ (PE_2_) release increased by about 4-fold, 30 min post-load; they also noted increased type I pro-collagen secretion for up to 5 days post-load. The secretion of these two agents is a clear indicator of bone formation, as PE_2_ is a crucial regulator of osteoblast proliferation and differentiation [146]. Additionally, type I pro-collagen synthesis has been correlated with bone collagen synthesis and bone formation rate [147].

## 5. Stimuli-Responsive dECM-Based Hydrogels for the Delivery of Therapeutics

**Figure 5:**
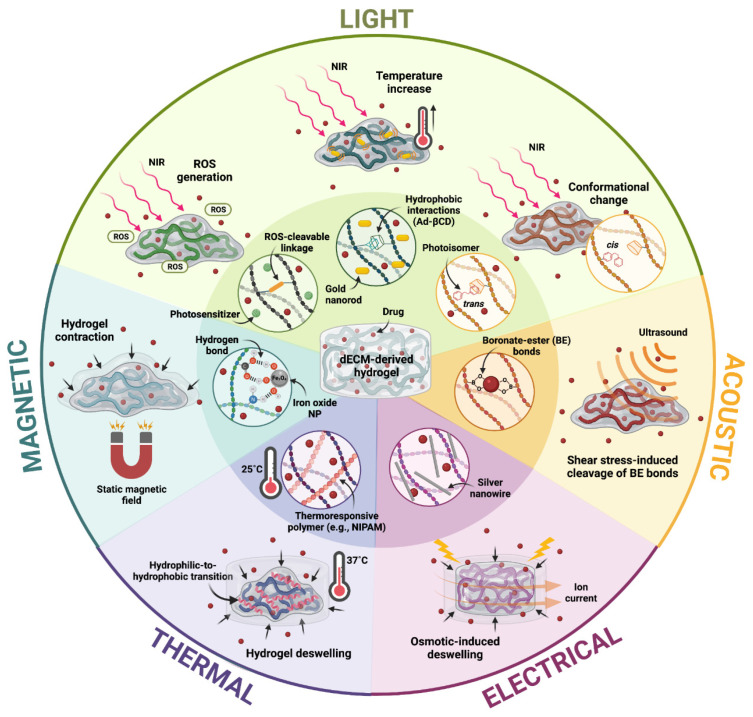
General schematic of drug release mechanisms from dECM-derived hydrogels induced by external stimuli, namely light, temperature, or magnetic, acoustic, and electric fields. Hydrogel destabilization, degradation, or deswelling as a consequence of these stimuli mediates the release profiles of encapsulated molecules or drugs (Created with BioRender.com).

Hydrogels have been widely implemented for the localized and controlled delivery of therapeutics due to their versatility and high biocompatibility. Several design parameters such as pore size, backbone charge, hydrophilicity, and crosslinking density, can be strategically tuned to modulate the solubility/dispersibility of hydrophobic and hydrophilic molecules within hydrogels [13]. Moreover, their release profiles can be precisely controlled with specific changes in hydrogel structure induced by swelling, dissolution, or degradation, as a consequence of exposure to external stimuli (e.g., light, ultrasound, and electric and magnetic fields) [148]. The strategic design of drug-loaded hydrogels has therefore been directed towards the inclusion of stimuli-responsive molecules (e.g., polymers, proteins and peptides) or nanostructured materials (e.g., iron oxide, gold nanoparticles, carbon nanotubes, graphene oxide, and reduced graphene oxide) that respond to individual or combined stimuli [15]. Recent approaches that have employed external stimuli for controlling drug release profiles in rationally designed dECM-derived hydrogels are discussed below and summarized in Figure 5.

### 5.1. Light-Triggered Drug Release

As evidenced in previous sections, the incorporation of light-sensitive nanostructured materials, molecules, or polymers into ECM-based hydrogels has been extensively investigated in recent years. However, the use of these hydrogels as vehicles for controlled drug delivery upon exposure to light is still largely unexplored.

As NIR laser irradiation is undoubtedly the most extensively studied light stimulus for controlled drug delivery on ECM-based hydrogels, a broad variety of release mechanisms triggered by this stimulus have been considered in several pharmacological applications. NIR-triggered photodynamic therapy (PDT), a process in which cell death is induced by an increase in ROS generation with the activation of a photosensitizer, is one of the emerging anticancer therapies with the highest potential for clinical translation [149]. This is as, through this approach, it is possible to simultaneously harness the controlled activation of anticancer drugs and their release rates from the hydrogel matrix. For example, Xu and colleagues relied on the elevated levels of ROS during PDT to induce hydrogel degradation and the subsequent release of the chemotherapeutic drug doxorubicin (DOX) [43]. In this work, the researchers developed an injectable and photodegradable HA-based hydrogel loaded with DOX for achieving NIR light-tunable and on-demand drug release for combined PDT and chemotherapy. The developed hydrogel consisted of adipic dihydrazide-modified HA (HA-ADH) conjugated with the photosensitizer protoporphyrin IX (PpIX) (HA-ADH-PpIX). Moreover, a dialdehyde-functionalized thioketal (TK−CHO) crosslinker was incorporated into the hydrogel for introducing ROS-cleavable acylhydrazone bonds between the hydrazide groups of HA-ADH-PpIX and the benzaldehyde groups of TK-CHO. Upon irradiation with NIR light (633 nm), PpIX was activated, and the resulting high local production of ROS facilitated hydrogel degradation by cleaving the acylhydrazone bonds within the hydrogel network. The controlled release of DOX, combined with the induced cell death by the high ROS levels, demonstrated an outstanding anticancer activity in both an in vitro breast cancer model (MCF-7 cells) and an in vivo 4T1 tumor-bearing mouse model [43].

Alternatively, Xing and coworkers potentiated the anticancer effects of PDT with the localized release of NIR light-activated anticancer drugs and a local temperature increase [14]. They developed an injectable nanocomposite hydrogel by incorporating gold nanoparticles (AuNPs) within a collagen hydrogel loaded with the photosensitive drug meso-Tetra (N-methyl-4-pyridyl) porphine tetrachloride (TMPyP), which becomes cytotoxic and produces ROS upon NIR laser irradiation (635 nm). Moreover, the presence of AuNPs in the hydrogel permitted a simultaneous photothermal therapy (PTT), as these nanoparticles exhibit a high photon-to-heat conversion efficiency upon irradiation with NIR light. This led to a remarkable local temperature increase which, in combination with the cytotoxic effect of activated TMPyP, resulted in an exceptional antitumor efficacy in a breast (MCF-7) tumor-xenograft mouse model. In addition, the researchers demonstrated that the hydrogel significantly improved the bioavailability of the drug by protecting it from rapid body clearance, as well as minimized the collateral effects by allowing a localized retention of the treatment within the tumor region [14].

PTT alone has also been employed for controlling cargo release via degradation of hydrogel networks in response to local temperature increase. For instance, Highley and coworkers developed an NIR-responsive nanocomposite hydrogel by mixing gold nanorods with a β-cyclodextrin (βCD)- and adamantane (Ad)-modified HA hydrogel [150]. They exploited the light-absorbing properties of gold to induce local heating upon irradiation and, in turn, destabilize the hydrophobic interactions between βCD and Ad. This resulted in hydrogel degradation and subsequent payload release. Their results showed that NIR irradiation is well-suited to induce plasmonic heating and increase the release of encapsulated molecules between 0.37–500 kDa by more than 2-fold [150]. Alternatively, Sun and colleagues developed an NIR-responsive hydrogel based on benzoxaborole-modified HA (BOB-HA) and a fructose-based glycopolymer, supplemented with perylene diimide zwitterionic polymer (PDS) as photosensitizer, photothermal polymeric nanoparticles, ascorbic acid, and DOX [45]. By irradiating with NIR at 660 nm, PDS and ascorbic acid interacted for the conversion of oxygen into hydrogen peroxide, which was able to cleave dynamic covalent bonds based on benzoxaborole-carbohydrate interactions within the hydrogel network. This controlled degradation led to the site-specific release of the embedded photothermal nanoparticles and the DOX for chemotherapy. Moreover, upon irradiation with 915 nm NIR 3 days after the first irradiation, the photothermal nanoparticles were able to locally increase temperature for PTT. As a result, this combined therapy nearly eradicated tumors in a 4T1 mouse model, while those untreated kept growing.

Lastly, light stimuli have also been shown useful in drug delivery applications by inducing conformational changes in hydrogels. This is exemplified in ECM-based hydrogels with the work reported by Wu and colleagues [151]. Briefly, the authors developed NIR-responsive core-shell hybrid nanogels for fluorescence imaging and combined chemo-photothermal cancer therapy. The hybrid nanogels were assembled by coating Ag-Au bimetallic nanoparticles with a thermo-responsive nonlinear PEG-based hydrogel shell decorated with superficial semi-interpenetrating HA chains. The temperature increase in Au cores upon NIR irradiation, as well as the local temperature increase during incubation, induced a hydrophilic to hydrophobic transition of the PEG chains in the shell, which destabilized its interactions with the loaded anticancer drug (temozolomide) and allowed its release. These hybrid nanogels exhibited high anticancer activity against mouse melanoma B16F10 cells as it allowed a multimodal therapy that combined localized chemotherapy with NIR-triggered photothermal treatment [151]. Likewise, Rosales and colleagues designed a drug delivery system based on a UV light-responsive azobenzene- and βCD-modified HA hydrogel, crosslinked via azobenzene-βCD hydrophobic interactions. Azobenzene is a photoisomer that transitions from its trans to cis isomeric configuration upon exposure to light in the 350–550 nm wavelength range [152]. This transition induces a conformational change within the hydrogel that reduces azobenzene’s affinity for the hydrophobic cavity of βCD and, in turn, the reduced crosslinking density within the hydrogel allows the release of the encapsulated molecules. However, when the light stimulus is removed, the azobenzene moieties return to its *trans* state and the hydrogel recovers its original conformation. This reversible behavior grants a high tunability of its mechanical properties and makes it suitable for different applications, such as drug delivery or mechanobiology studies, in which temporal regulation of material properties is key for robust and more compelling studies [153].

### 5.2. Magnetic-Triggered Release

Magnetic forces, induced by static or dynamic magnetic fields, have been used to trigger the controlled release of molecules from hydrogel depots. This release can be mediated by reversible or non-reversible structural changes that disrupt the hydrogel network itself or the bonds that entrap the molecules within the hydrogel. Bettini and colleagues, for example, developed an ION-embedded collagen hydrogel scaffold with drug-retaining and paramagnetic properties [57]. IONs were covalently conjugated to the collagen network by implementing a dehydrothermal (DHT) treatment at 120 ºC for 48 h. As controls, the authors synthesized collagen hydrogels without IONs and crosslinked either by a chemical treatment with formaldehyde and DHT or solely by DHT treatment. Surprisingly, ION-conjugated hydrogels exhibited a higher crosslinking degree, as demonstrated by smaller pore size and a lower swelling degree. Moreover, only the ION-conjugated and formaldehyde-crosslinked hydrogels were capable of retaining loaded cargo, i.e., fluorescein molecules. After stimulating the loaded ION-conjugated hydrogels with a permanent magnet, deformation of the scaffold was induced, thus facilitating the flow of water and dissolved fluorescein out of the gel by means of pore collapse. By applying consecutive magnetic stimulation cycles, they demonstrated the controlled release of fluorescein.

Similarly, Dai and colleagues proposed dopamine-conjugated hyaluronan (HA-DOPA) hydrogel embedded with IONs as a drug delivery platform for anticancer therapies [56]. In this hydrogel, crosslinking was facilitated by the interaction between catechol groups of dopamine and iron(III) of IONs, and DOX was then loaded and retained by means of electrostatic interactions with the positively charged HA-DOPA. Moreover, besides serving as crosslinking agents, IONs also played an important role in the on-demand release of DOX. The authors demonstrated that the release rate of the entrapped drug could be accelerated with an external alternating magnetic field (AMF), as it generated local hyperthermia and subsequent hydrogel destabilization. The authors also showed that the release rate could be slowed down when removing the applied magnetic stimulus. In vivo experiments were performed on a A375-xenografted tumor mice model, where the combined chemotherapy (DOX) and hyperthermia (IONs) therapy resulted in significantly smaller tumor volume after 18 days when compared to the control group treated only with PBS. Moreover, single modality treatment (either chemotherapy or hyperthermia) failed to show an equivalent efficacy of the combined therapy at the same dose level of the chemotherapeutic agent.

### 5.3. Ultrasound-Triggered Release

Mechanical stress generated by ultrasound has also been employed to disrupt the bonds between drugs and hydrogels for enabling drug release. Sun and coworkers designed a dual-crosslinked hydrogel based on methacrylic HA and a four-armed PEG acrylate (4arm-PEG-Aclt) for the ultrasound-induced delivery of tannic acid as a drug model [154]. The crosslinked hydrogel network was constructed through free radical polymerization of 4arm-PEG-Aclt with HA previously modified with 4-(aminomethyl) phenylboronic acid and methacrylic anhydride (PhB-mHA). Then, a dynamic crosslinking was achieved by forming boronate ester bonds between phenylboronic acid (conjugated along the polymeric network) and tannic acid. This dual-crosslinking endowed the hydrogel with remarkable mechanical properties, thereby making it resistant to deformation by external mechanical forces (i.e., compression). Moreover, boronate ester bonds dynamically respond to ultrasound, thereby allowing the non-invasive and on-demand release of tannic acid. This can be attributed to the dynamic shear force generated by this stimulus, which is enough to disrupt such bonds [154].

Alternatively, ultrasound-induced drug release has been achieved by destabilizing encapsulating agents with acoustic droplet vaporization (ADV) mechanisms, which mediate their transition from liquid to gas phase in response to pressure induced by acoustic stimuli. In this regard, Dong and colleagues developed an acoustically responsive hydrogel consisting of sonosensitive micron-sized emulsions embedded within a fibrin matrix which, under 2.5 MHz sound waves, released encapsulated basic fibroblast growth factor (bFGF) [58]. The emulsion entails a water-in-perfluorocarbon (PFC)-in-water (W_1_/PFC/W_2_) double emulsion, with bFGF in the W_1_ phase, and its release occurs when PFC within each droplet phase transitions from liquid to gas, thereby disrupting the droplet morphology. They showed that this hydrogel-emulsion system allowed a non-invasive and on-demand release of bFGF, as hydrophobic PFC acted as a diffusion barrier for bFGF until its removal by ADV. To further demonstrate the applicability of this hydrogel-emulsion system, they added an external layer of a HUVEC-laden fibrin hydrogel to test whether angiogenesis is promoted by bFGF release. In this regard, HUVEC angiogenic sprouting was significantly enhanced after ADV-mediated bFGF release, specifically when increasing acoustic pressure and emulsion volume fraction. A recent work by the same group demonstrated that release profiles of two or more payloads could be specifically modulated by loading them within emulsion droplets containing PFCs with different ADV thresholds (i.e., with varying carbon chain lengths in the PFC) [59]. Accordingly, by tuning the frequency of ultrasound standing wave fields (SWFs), they could precisely control the release of two different factors relevant in angiogenesis, namely bFGF and platelet-derived growth factor BB (PDGF-BB), at specific time points during maturation.

### 5.4. Electric-Triggered Release

Although electrical stimuli have been largely unexplored for controlling drug release in dECM-derived hydrogels, a recent work by Ha and colleagues harnessed the ionic currents induced by this type of stimulus for modulating release profiles of model drugs within collagen-GelMA hydrogels embedded with silver nanowires (AgNWs) [53]. They showed that when applying a current with electric potential of 1V, a consistent weight loss was observed as a function of exposure time in the conductive hydrogel, while collagen and GelMA hydrogels remained unchanged. They attributed this weight loss to the enhanced electrical conductivity of AgNW, which facilitated the generation of an outward osmotic gradient as a consequence of the flow of ions crossing the hydrogel. Moreover, this weight loss was highly correlated to the release of encapsulated fluorescein isothiocyanate (FITC)-dextran particles, which suggests that electrically induced structural changes and promote outward flows of fluids that facilitate the release of loaded molecules [53].

### 5.5. Temperature-Triggered Release

Temperature-responsive materials have also been incorporated within dECM-hydrogels for directing the release of loaded cargoes as most delivery applications must be performed at physiological temperature (37 °C). As an example, Ravichandran and colleagues described the synthesis of a temperature-responsive injectable hydrogel for the controlled release of drugs and proteins, based on ColMA building blocks and the thermo-responsive polymer N-isopropyl acrylamide (NIPAm) [155]. In this case, drug release is attributed to temperature-induced hydrogel deswelling, as well as changes in the drug/molecule affinity to the matrix, as NIPAm is hydrophilic at 25 °C but becomes hydrophobic at 37 °C. In consequence, as temperature approaches physiological conditions, the hydrogel shrinks due to conformational changes from elongated to coiled morphologies, which induces an outward flow of water and alters drug-hydrogel affinity. Moreover, hydrogel collapse is prevented due to the pH-responsiveness of ColMA. As carboxyl groups of collagens are converted into carboxylate anions (–COO^−^) at physiological pH (~7.4), the electrostatic repulsion between them grants sufficient hydrogel porosity for facilitating drug release. The coupled effect of NIPAm solubility change and ColMA swelling favored significantly the release of the model drugs bovine serum albumin (BSA) and vitamin E, yielding over 80% release after 5 days [155].

## 6. Perspectives on Clinical Translation

Despite the considerable advances in the engineering of functional ECM-based hydrogels, their translation into clinical scenarios is still in its infancy. In particular, few preclinical models have been developed for evaluating their performance beyond in vitro setups. ECM-based hydrogel constructs destined for replacing injured tissue or organ defects have been limited to in vitro characterizations and monitoring of their maturation dynamics, mainly as true biomimicry has not been fully accomplished yet. Accordingly, only a handful of these hydrogels have been implanted in animal models, and these usually comprise simple cellular patterns that can be easily integrated with host tissues [105]. This is the case of the HA-based constructs developed by Kang and colleagues, in which acoustically patterned cells unidirectionally aligned in cylindrical tubes were able to integrate with mouse vasculature and form perfusable and interconnected networks [105]. Similarly, of the thirteen works mentioned in Section 5 where ECM-based hydrogels have been implemented for the localized delivery of therapeutic agents, only four have evaluated drug release dynamics and effects on animal models: [14,43,45,56]. This, in turn, could have been the result of the low outreach and reception that ECM-based hydrogels have had on drug delivery fields, where highly accessible and easier to use synthetic or natural biodegradable materials are commonly employed [156].

Moreover, according to the ClinicalTrials.gov database by the U.S. National Library of Medicine, there has been only one clinical trial where tissue-derived ECM hydrogels have been studied. This trial was conducted in the U.S., and its main aim was to evaluate the safety and feasibility of a porcine myocardial tissue-derived ECM hydrogel delivered trans-endocardially to human subjects following myocardial infarction [157]. Although the study was not intended to evaluate efficacy, physicians reported myocardial function improvement for all intervened patients (*n* = 15). This clinical trial demonstrated the significant advances that the tissue engineering industry and academic research groups have accomplished towards overcoming translational challenges faced by tissue-derived ECM hydrogels, particularly in terms of shelf-life and scalability [19]. It also further demonstrated the biomimetic potential of ECM-based hydrogels that could be further exploited for facilitating the translation of TE and RM into clinical scenarios.

The translation of ECM-based hydrogels into clinical settings is especially appealing, as they hold much promise for the emerging field of precision medicine. For instance, Noor and colleagues demonstrated the feasibility of personalized regenerative medicine by developing cardiac patches using patient-derived ECM hydrogels and cells [158]. From a biopsy of omental tissue, the authors isolated and reprogramed the patient’s own cells into stem cells, while the ECM was processed into a personalized hydrogel. The reprogrammed stem cells were further differentiated into cardiomyocytes and endothelial cells, embedded into either the ECM hydrogel or a sacrificial material, and bioprinted into thick and perfusable cardiac patches. Although they only addressed the formulation and bioprinting of these constructs, the diverse strategies described in this review for improving tissue organization and morphogenesis could be exploited for improving construct maturation, taking the field one step closer towards the biofabrication of functional personalized tissues suitable for transplantation. Alternatively, the isolation and biointegration of diseased human cells into ECM-derived hydrogels could potentiate the development of in vitro models for studying the involved physiological and pathophysiological hallmarks. Goldfracht and colleagues, for example, showed that in vitro heart tissue models with patient-derived hiPSC-cardiomyocytes embedded in ECM-based hydrogels (discussed in detail in Section 4.3) could recapitulate the abnormal phenotypes of arrhythmogenic disorders by using diseased cells from patients [143]. They demonstrated that this disease model could be used even further to create a personalized model to evaluate the efficacy of therapeutics against patient-specific cardiac pathologies. The implementation of biofabrication technologies appears critical for developing robust in vitro models of disease that could be eventually translated into the clinical practice for the high-throughput screening of therapeutics. We believe that this is a route that enables a foreseeable future of favorable outcomes for diseased patients.

## 7. Concluding Remarks

ECMs have been devised as the next-generation materials for alleviating the limitations regarding functionality and cell development faced with advanced TE technologies, such as 3D bioprinting and RM therapies. The utilization of these materials for the formulation of hydrogels is considered one of the most promising approaches for achieving superior tissue functionality due to their inherent biochemical composition that can guide cell thrive. However, their heterogeneous and biomimetic biochemical composition alone has been demonstrated insufficient for guaranteeing morphogenesis of engineered tissues in vitro or in vivo, nor to be appropriate for their use in advanced biomanufacturing technologies that require particular mechanical properties.

As shown in this review, external stimuli have been pivotal for facilitating the recapitulation of biologically relevant micro- and macro-environments in bioengineered tissues. For instance, light stimuli between the UV and visible ranges have become the gold standard for facilitating crosslinking schemes that ultimately improve the mechanical properties of ECM-based hydrogels. Directed electric, magnetic and pressure fields have been crucial for sculpting microarchitectural characteristics that drive cellular organization in tissue-specific patterns. Their effects on electrochemical gradients have been fundamental, as well, for directing stem cell differentiation profiles and dictating morphogenetic cues that mediate tissue maturation. Beyond their contribution in the biofabrication of biomimetic constructs, external stimuli have proven to be critical for controlling the delivery of therapeutics from hydrogel depots. This comprises an advanced strategy for accelerating tissue regeneration, both in vitro and in vivo, with specific release profiles of embedded biomolecules or drugs.

However, this review demonstrates that among the limited collection of papers that developed and evaluated stimuli-responsive hydrogels based on ECM components, only a handful really implemented tissue-derived ECMs as their main hydrogel component. Collagen and HA hydrogels are undoubtedly the most commonly used for implementing the referred schemes, probably due to their accessibility and ease of use. Nevertheless, we believe that the synergistic action of the native ECM components in tissue-derived ECM hydrogels, coupled with the mentioned stimulation regimes, could significantly potentiate these results, leading us one step closer towards functional and biomimetic constructs for TE and RM.

## Figures and Tables

**Table 1:** Most relevant works implementing external stimuli for improving cellular scaffolding and localized drug delivery from dECM-derived hydrogels.

Stimulus	Stimulation Parameters	Stimulus Enhancer	Hydrogel	Embedded Cells/Cargo	Effect	Ref.
Light	UV light (365 nm) at 18 W/cm^2^ intensity for ‘several’ seconds.	Irgacure 2959	Porcine liver dECM, thiolated-HA, thiolated-gelatin, PEG-acrylate, and PEG-alkyne	Primary human hepatocytes, primary human stellate cells, and primary human Kupffer cells spheroids.	Bioink stiffness increases by more than one order of magnitude upon light stimulus.	[34]
	White light exposure for 5 min post-bioprinting.	Eosin Y	Cardiac dECM-GelMA	Neonatal human cardiac progenitor cells	Improved mechanical stiffness upon photocrosslinking while maintaining cell viability above 75%.	[35]
	Blue-light exposure (405 nm) with 1.5 W/cm^2^ intensity for 40 s.	Riboflavin phosphate (RFP)	Thiol- and methacryloyl-modified HA	Primary rabbit corneal fibroblasts	Improved hydrogel crosslinking and mechanical stiffness in an RFP-dependent manner. Crosslinking degree dictated bovine serum albumin release profiles and hydrogel degradation.	[36]
	NIR laser excitation (808 nm) at 5.6 and 8.3 mW/cm^2^ power densities for 3 min every other day	Carbon dot nanoparticles	Type I collagen	Bone marrow-derived stem cells (BMSCs)	Increased proliferation and chondrogenic differentiation of BMSCs as a result of non-lethal doses of ROS produced with photodynamic therapy.	[37]
	UV exposure (365 nm) for 120 s.	Irgacure 2959	Methacryloyl-modified kidney dECM, HA, gelatin, and glycerol	Human primary kidney cells	Almost 2-fold increase in storage modulus after photocrosslinking when compared to unmodified kidney dECM-based hydrogels.	[38]
	Blue light exposure (405 nm) at 20 W/cm^2^ for 15, 30, and 45 s.	LAP	Methacryloyl-modified bone dECM	Human dental pulp stem cells, hMSCs, and HUVECs	Storage modulus increase in a dose-dependent manner.	[39]
	Blue light exposure (405 nm) at 62 W/cm^2^ for 1 min	Riboflavin	Methacryloyl-modified SIS dECM	Adipose-derived MSCs	Two-fold increase in storage modulus upon photocrosslinking.	[40]
	Blue light exposure (405 nm) at 30 mW/cm^2^.	Ruthenium/sodium persulfate	Corneal and heart dECM	Human bone marrow- and turbunate-derived MSCs, hiPSC-derived cardiomyocytes	A 2.55- and 3.79-fold increase in compressive and storage modulus, respectively, upon photocrosslinking. High shape fidelity allowed bioprinting of multi-layered and complex anatomical structures.	[41]
	Blue light exposure (laser at 488 and LED at 460 nm).	tris (2,2′-bipyridyl) dichlororuthenium (II) hexahydrate/sodium persulfate.	Fibrin	Normal human dermal fibroblasts	The stiffness of fibrin hydrogels was patterned at the micron scale by spatio-selective irradiation with a blue light laser.	[42]
	NIR laser irradiation (635 nm) at 169.85 mW/cm^2^ for 10 min.	meso-Tetra (N-methyl-4-pyridyl) porphine tetrachloride (photosensitive drug) and gold nanoparticles	Type I collagen	meso-Tetra (N-methyl-4-pyridyl) porphine tetrachloride	After a single injection, but multiple treatments with NIR, the combined photothermal and photodynamic therapies achieved complete tumor eradication on a breast tumor xenograft mice model.	[14]
	NIR laser irradiation (633 nm) at 50 mW/cm^2^ for 10 min.	Protophorphyrin IX (PpIX)	Adipic dihydrazide-modified hyaluronic acid conjugated with PpIX, and dialdehyde-functionalized thioketal containing a ROS-cleavable thioketal linker	Doxorubicin (DOX)	Combined photodynamic therapy and chemotherapy that nearly suppressed tumor growth on a tumor xenograft mice model.	[43]
	NIR laser irradiation (760 nm) at 0.47 W/cm^2^ for 3 min.	Indocyanine green	Type I collagen, poly (gamma-glutamic acid)	DOX or granulocyte macrophage colony-stimulating factor	Upon local temperature increase induced by NIR irradiation, the hydrogel network was disrupted, and payload release was increased up to 3-fold in vitro.	[44]
	NIR laser irradiation at 660 nm (0.5 W/cm^2^) for 20 min and, three days later, at 915 nm (0.5 W/cm^2^) for 10 min.	Perylene diimide zwitterionic polymer	Benzoxaborole-modified HA, fructose-based glycopolymer	DOX and photothermal polymeric nanoparticles	In a 4T1 tumor bearing mice model, tumors were almost eradicated with a combined chemo- and photothermal therapy. First, NIR irradiation at 660 nm led to enzymatic degradation of the hydrogel, thus releasing the DOX and the photothermal nanoparticles. Then, after 3 days, a second NIR irradiation, but at 915 nm, led to localized hyperthermia for killing tumor cells.	[45]
Electric	Biphasic waveforms with 50 ms pulses of 3–5 V/cm at 0.5, 1, 2, and 3 Hz.	rGO	GelMA	Primary neonatal rat cardiomyocytes	Increased contractility of rGO-GelMA constructs with respect to GelMA.	[46]
	(i) Dielectrophoresis before crosslinking: sinusoidal electric field of 1 MHz and 20 V for 10 s. (ii) Biphasic waveforms with 10 ms pulses of 3 V at 1 Hz applied continuously from day 2 to day 4.	MW-CNTs	GelMA	129/SVE-derived mouse stem cells (embryoid bodies)	Enhanced electrical conductivity, mechanical stiffness, and cardiac differentiation in aligned CNT-GelMA constructs when compared to unaligned CNT-GelMA and pristine GelMA.	[47]
	Rectangular electrical pulses of 2 ms at 1, 2, and 3 Hz applied continuously from days 3 to day 7.	Dopamine-rGO	GelMA	Cardiomyocytes	Improved orientational order of sarcomeres, propagation of intercellular pacing signals, and calcium handling.	[48]
	Square-wave pulses of 5 V at 1.5 Hz.	rGO	Myocardial dECM	hiPSC-derived cardiomyocytes	Improved electrophysiological function (calcium handling, action potential duration, and conduction velocity) and physiologically relevant drug responses in rGO-GelMA.	[49]
	Constant electric field of 100 mV/cm applied 20 min daily for 1 week.	Polydopamine-modified black phosphorus (PDA-BP) nanosheets	GelMA	Rat bone marrow derived MSCs	Enhanced neural differentiation of MSCs in stimulated PDA-BP@GelMA hydrogels when compared to unstimulated PDA-BP@GelMA and GelMA.	[50]
	External electric field of 5 V AC pulses at 1 Hz applied continuously for 21 days.	GNWs	Type I collagen	C2C12 myoblasts	Aligned GNWs in electric field direction favored myoblast alignment and enhanced myotube formation.	[51]
	DC stimulation at 50 mV/mm applied continuously for 8 hrs.	SW-CNTs	Matrigel-type I collagen	Neonatal rat dorsal root ganglia cells	Enhanced neurite outgrowth on stimulated SWCNT-loaded hydrogels when compared to unstimulated SWCNT-loaded hydrogels and SWCNT-free hydrogels.	[52]
	Electric current below 1 mA generated with an electric potential of 1 V. Applied for 15, 30, 45, and 60 min.	Ag nanowires	GelMA-Collagen	Fluorescein isothiocyanate (FITC)-dextran	Ion currents during electrical stimulation created osmotic gradients that caused periodic hydrogel contractions and facilitated drug release.	[53]
Magnetic	2 mT cylindrical magnet placed beneath the constructs.	Streptavidin-coated IONs	Type I collagen-agarose	Human primary knee articular chondrocytes	Collagen fiber alignment in magnetic field direction, as a result of ION motion inside hydrogel, improved type II collagen secretion by chondrocytes.	[54]
	100 mT magnetic field.	Rod-shaped acrylate-modified poly(ethylene oxide-stat-propylene oxide) microgels embedded with IONs	Fibrin	Chicken-derived primary dorsal root ganglions	Unidirectional alignment of rod-shaped microgels in field direction-oriented neurite outgrowth inside hydrogel.	[55]
	External alternating magnetic field (1478 Hz, 10 A, 10 min).	IONs	Dopamine-conjugated hyaluronan and IONs	DOX	IONs serve as structural crosslinkers and facilitate hyperthermia treatment and on-demand release of DOX under alternating magnetic fields.	[56]
	Permanent magnet placed against the hydrogel immersed in water.	IONs	Collagen	Fluorescein sodium salt	Cargo release was triggered and accurately controlled upon hydrogel deformation induced by external magnetic field.	[57]
Acoustic	2.5 MHz ultrasound at either 3.3 or 8.8 MPa peak rarefactional pressures with 4.3 or 17.2 MPa peak compressional pressures, respectively.	Perfluorocarbon (PFC) double emulsion droplets	Fibrin	bFGF	Ultrasound stimulus induced vaporization of PFC in the double droplet emulsions, thereby causing the release of encapsulated bFGF. The timely release of this factor promoted angiogenic sprouting of HUVECs within the outer layer of the fibrin hydrogel.	[58]
	Acoustic standing wave fields of 3.25 MHz at day 0 and of 8.6 MHz at day 4	Two PFC double emulsion droplets based on perfluoropentane and perfluorohexane	Fibrin	bFGF and PDGF-BB	Sequential payload release induced by vaporization of PFC double droplet emulsions at different pressure thresholds modulated with ultrasound standing wave fields. bFGF was released at day 0 and PDGF-BB at day 4.	[59]
